# Estimating the Effect of Physical Education Reform on College Students’ Mental Health with Difference-in-Differences

**DOI:** 10.3390/bs16101944

**Published:** 2026-10-19

**Authors:** Zheng Ma, Ran Zhu, Galiya Madiyeva, Aigul Bulatbayeva

**Affiliations:** 1 Faculty of Medicine and Health, AL-Farabi Kazakh National University, Almaty 050040, Kazakhstan; ma_chzhen@live.kaznu.kz (Z.M.); galiya.madiyeva@kaznu.kz (G.M.); 2 Faculty of Physical Education, Huanggang Normal University, Huanggang 438000, China; 3 Faculty of Philosophy and Political Science, AL-Farabi Kazakh National University, Almaty 050040, Kazakhstan; bulatbaeva.aygul@kaznu.kz

**Keywords:** propensity score matching, difference-in-differences, mental health, college physical education, college students

## Abstract

Facing increasingly fierce competition, mental health issues among college students have become a growing concern in higher education. College physical education (PE) plays a vital role in improving mental health among students. However, its causal effects and potential mechanisms remain insufficiently studied. In this study, we use a difference-in-differences (DID) model to investigate the impact of PE reform on college students’ mental health. To address individual heterogeneity, common time trends, and potential sample selection bias, the propensity-score matching DID (PSM-DID) method was applied. We analyzed 3066 student–wave observations from 1533 unique students at universities in 26 Chinese cities during 2021–2024; each student was observed in two survey waves. The intervention was a one-year compulsory PE reform involving adjustments to course design and expanded opportunities for physical activity. Students attending reform-adopting universities in 15 cities constituted the treatment group, while students attending universities without the reform in 11 cities formed the comparison group. A time-varying difference-in-differences model, supplemented by propensity-score matching, examined changes in CES-D-10 depressive-symptom scores using city-level reform adoption dates. Results show college PE reform significantly reduced CES-D scores. In the fully adjusted university- and wave-fixed-effects model with city-clustered standard errors (26 cities), PE reform was associated with a 1.22-point reduction in CES-D scores (β = −1.22; SE = 0.38; 95% CI: −1.96 to −0.49; wild cluster bootstrap *p* = 0.019). Excluding self-rated mental health left the estimate essentially unchanged (β = −1.24). On the matched sample, with SH omitted from the propensity-score model, the corresponding estimate was β = −1.28 (N = 1974). A formal interaction test indicated a larger effect in first-tier cities (*p* = 0.014); academic-year differences were jointly significant (*p* = 0.043). Mechanism analysis further indicates that PE reform was also associated with increased exercise habit intensity and exercise frequency, suggesting potential behavioral channels. This study shows benefits of college physical education reform for mental health and offers important policy implications.

## 1. Introduction

In modern society, college students face increasing pressure from multiple aspects, including academic pressure, interpersonal relationship pressure, and pressure regarding future career planning (Hariharan et al., 2025). Symptoms such as anxiety and depression have been on the rise among students, posing new challenges to higher education institutions in promoting the overall well-being of students (Salimi et al., 2023). Although universities have strengthened psychological counseling services and mental health education programs, and many students are able to cope with stress through self-regulation or professional support, the risks remain an urgent problem to be solved (Lei et al., 2021).

As a positive and healthy lifestyle, regular physical exercise has been proven to reduce stress, regulate emotions, enhance self-efficacy, and improve social interaction (Mahindru et al., 2023). In recent years, physical education (PE) courses have been incorporated into the higher education curriculum system in China and have gradually become compulsory courses for college students (Du & Liu, 2022). Through systematic PE courses, students can acquire scientific sports knowledge, develop good exercise habits, enhance physical and mental resilience, and cultivate self-discipline, cooperation, and perseverance through participation in group activities and rules (Andermo et al., 2020). It plays an irreplaceable role in promoting physical and mental health and all-round development. These studies provide relevant background but do not directly establish whether changes in compulsory university PE provision reduce depressive symptoms (Schuch et al., 2016). The question addressed here is whether students in reform-adopting universities experience different changes in CES-D scores from students in comparison universities. We examine this question using variation in reform adoption timing and explore exercise habits and exercise frequency as potential behavioral channels.

Difference-in-differences (DID) is a quasi-experimental approach to policy evaluation that accounts for time-invariant unobserved differences (Arkhangelsky et al., 2021). With staggered adoption, comparison-group selection and treatment-effect heterogeneity require particular attention (Callaway & Sant’Anna, 2021; Goodman-Bacon, 2021). DID has been applied across policy settings, including labor markets (Johnston & Makridis, 2025) and educational interventions (Corral & Yang, 2024; Högberg & Horn, 2022). The simple one-way differential method, which relies solely on time comparisons, can yield biased results. Changes in students’ mental health are often influenced by a variety of factors, such as academic pressure, social or environmental shocks, adjustments to the curriculum, and the implementation of other educational or mental health policies. Students in colleges that have not implemented PE reforms may also experience similar psychological changes due to external factors. Without accounting for these confounding factors, it may overestimate or underestimate the real effectiveness of PE. To overcome this challenge, we employ a multi-period DID combined with propensity-score matching (PSM-DID) to identify policy effects. The intervention involved a one-year compulsory PE program aimed at encouraging regular physical activity, strengthening exercise habits, and supporting students’ physical and mental well-being. Its main components included adjustments to course design and expanded opportunities for physical activity. These changes included broader activity choices, progressive tasks adapted to students’ fitness levels, and greater emphasis on attendance, sustained participation, and individual progress in assessment. These changes may strengthen exercise habits and increase exercise frequency, providing potential behavioral pathways to improved mental health, consistent with meta-analytic evidence on exercise and depressive symptoms. We therefore examine these exercise behaviors as potential channels. Three main contributions of our paper are shown as follows.

(1) A multi-period DID framework is used to accommodate phased implementation of PE reform. This specification accommodates staggered adoption; causal interpretation remains subject to the identifying assumptions.
(2) By combining PSM, this model mitigates potential selection bias. It enhances the robustness and credibility of the empirical results.
(3) This work proposes using exercise habit intensity and exercise frequency as potential behavioral variables to examine whether PE reform is associated with changes in exercise behaviors.

## 2. Related Works

### 2.1. Role of College Physical Education

College PE combines physical activity with instruction and organized interaction among students and teachers. Reviews of PE and school sport discuss potential psychological and social benefits (Bailey et al., 2009), while college PE research considers students’ task values, perceived costs, and activity choices (Chen & Liu, 2009). These perspectives are relevant to the reform’s adjustments to course design and participation opportunities: accessible, engaging instruction may encourage sustained exercise, while group activities may provide opportunities for social interaction and perceived mastery. College PE represents the highest levels of school PE and is important in promoting lifelong participation in sports activities (Baena-Morales & González-Víllora, 2023). For scientific development, college PE should occupy an appropriate and clear position, fully reflecting its educational and competitive aspects. From an educational perspective, college PE consists of the entire education system, and its goals should be consistent with the overall goals of higher education (Brynteson & Adams, 1993). From a perspective of sports science, it is key in PE to emphasizing physical movement and exercise as a basic means.

College PE reform in China should be understood within the national policy framework for strengthening school physical education. The 2020 *Opinions on Comprehensively Strengthening and Improving School Physical Education in the New Era* further emphasizes improvements in PE teaching, exercise participation, and institutional support (General Office of the CPC Central Committee & General Office of the State Council, 2020). These documents provide a policy rationale for improving university PE provision.

### 2.2. Impact of PE on Student Mental Health

Research on effects of physical exercise on mental state and personality traits has focused primarily on comparing long-term activities with single-session exercise (Fuchs et al., 2011). Long-term physical exercise typically is regular or daily physical activity that lasts for a considerable period of time, with empirical studies generally suggesting at least one month. In contrast, single-session exercise refers to a short-duration, one-time physical activity. For terminology, physical activity (PA) refers to bodily movement that requires energy expenditure. Physical exercise is a subset of PA that is planned, structured, and repetitive and undertaken to maintain or improve physical fitness. Physical education (PE) refers to an organized educational process involving instruction, learning, and participation in movement and exercise. In this study, PE reform denotes the institutional intervention, whereas exercise habit intensity and exercise frequency represent students’ exercise behaviors. PE is an educational intervention that combines physical activity with instruction, organized participation, and interaction with teachers and peers. Consequently, evidence about exercise alone provides only a partial basis for understanding how changes in PE provision may affect mental health. Bailey et al. (2009) review the educational benefits attributed to PE and school sport, providing a framework for considering their psychological and social dimensions. Du and Liu (2022) specifically examine college PE and student mental health, while Andermo et al. (2020) synthesize evidence on school-related physical activity interventions and mental health among children. The latter offers relevant educational context, although its younger population limits direct application to university students. These studies motivate examining whether changes in PE provision influence depressive symptoms through structured exercise opportunities and the social and instructional environment. However, participation in exercise, exposure to PE, and institutional PE reform are distinct exposures. The present study therefore evaluates the reform itself, using evidence on physical activity to inform potential behavioral explanations rather than treating that evidence as direct proof of reform effectiveness.

Short-duration physical activity has been shown to significantly reduce negative emotions while maintaining high energy (Brisswalter et al., 1997). Even a single session of moderate-intensity exercise, such as cycling or running, can temporarily reduce anxiety levels in both healthy and unhealthy individuals (Zhu & Li, 2022). However, these psychological benefits are mostly temporary, and single-session exercise does not appear to provide sustained regulation of negative emotions. Without consistent participation, these positive effects on mental health tend to diminish over time. Long-term, regular physical exercise has more stable and lasting benefits for mental health (Mak et al., 2017). Aerobic exercise, rhythmic continuous exercise, and strength and flexibility training all contribute to improved mental health (Borland et al., 2022). While aerobic exercise is generally associated with significant improvements in physical fitness, anaerobic exercise can also provide similar mental health benefits. Long-term exercise has also been found to reduce anxiety levels and enhance self-efficacy, especially in people under high stress (Yeung, 1996). The psychological benefits of exercise are not solely dependent on improvements in physical function. Duration and regularity of exercise appear to be key factors in the positive role of consistent physical activity in promoting mental health (Huang et al., 2024).

Exercise duration exceeding a certain threshold is more effective in reducing anxiety, while exercise lasting for several weeks is crucial for alleviating more stable anxiety symptoms. Regular and long-term physical activity is vital in improving mental health and promoting mental well-being.

### 2.3. Difference-in-Differences Models and Theoretical Framework

DID models compare changes in outcomes between treated and comparison groups before and after an intervention (Dimick & Ryan, 2014). Variation in college PE reform adoption (Roth et al., 2023) motivates this approach, although causal interpretation requires credible parallel trends and appropriate treatment of staggered timing, comparison-group selection, and treatment-effect heterogeneity (Reingewertz, 2020). Individual and time fixed effects account for time-invariant individual differences and common period shocks, but do not eliminate differential time-varying confounding. Educational applications illustrate both the usefulness and limitations of DID. Contini and Cugnata (2020) highlight measurement-comparability problems in analyses of educational inequality, and related educational studies examine de-tracking reforms and educational aspirations (Holtmann et al., 2024) and cancer prevention education among college students (Jia et al., 2024). DID has also been applied in other policy evaluations (Blake et al., 2024; Eun, 2025; Rose et al., 2023; Schwartz et al., 2021).

The reviewed literature provides evidence on the mental health benefits of exercise and the educational value of PE, but offers limited direct evidence on whether institutional PE reform reduces depressive symptoms among university students. Exercise interventions and school-based studies do not fully capture changes in compulsory university PE provision. This study addresses this gap by estimating the effect of PE reform on depressive symptoms and examining exercise habits and exercise frequency as potential behavioral channels.

The proposed pathway is that revised PE provision expands structured participation opportunities, encouraging stronger exercise habits and more frequent exercise. These changes may support emotional regulation, perceived mastery, and social interaction, potentially reducing depressive symptoms. Accordingly, the reform coefficient in the CES-D model is hypothesized to be negative, while the reform coefficients in the exercise-habit and exercise-frequency models are hypothesized to be positive. These estimates assess the overall reform effect and its effects on potential channels.

## 3. Methods

**Figure 1:**
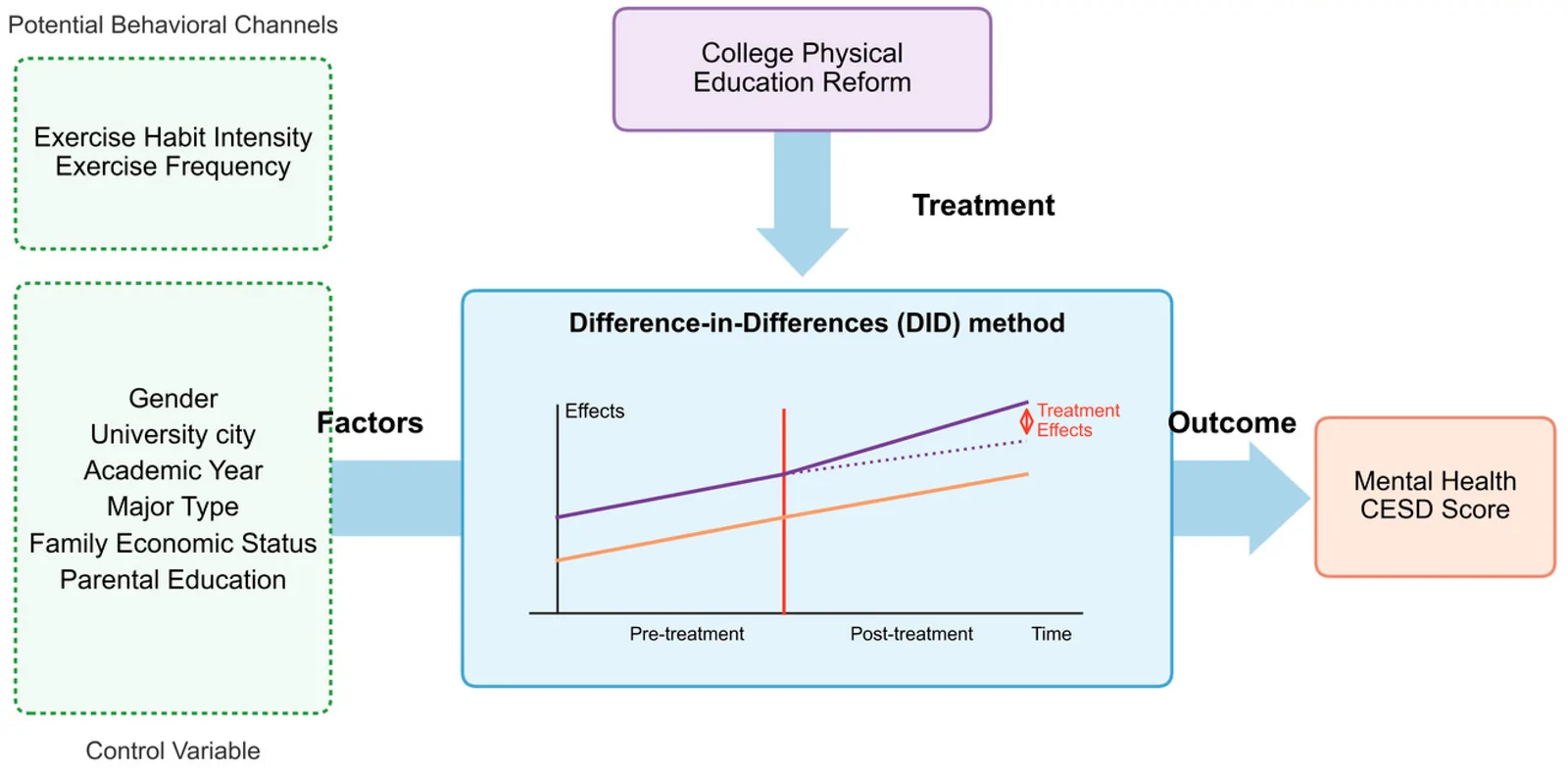
The overall workflow of the PSM-DID (multi-period DID) model.

Our DID method provides a more rigorous framework for identifying the effects of college PE reform on students’ depressive symptoms. The intervention involved a one-year compulsory PE program aimed at encouraging regular physical activity, strengthening exercise habits, and supporting students’ physical and mental well-being. Its main components included adjustments to course design and expanded opportunities for physical activity. Compared with the previous curriculum, the reform broadened activity choices to include aerobic exercise, team sports, and strength and flexibility training, introduced progressive tasks adapted to students’ fitness levels, and placed greater emphasis on attendance, sustained participation, and individual progress in assessment. The overall workflow is shown in Figure 1.

### 3.1. Time-Varying DID Model

Considering that college PE reforms are typically implemented in phases through policy pilots or course adjustments, the timing of reform adoption varies across universities and student cohorts. Unlike the traditional DID approach, which assumes a common policy implementation time, the time-varying DID model (Zeldow & Hatfield, 2021) accommodates heterogeneity in policy adoption timing and more accurately captures the dynamic effects of PE reform across institutions and periods, thereby avoiding estimation bias arising from a uniform timing assumption.

The timing of PE reform adoption varies across participating institutions, motivating a staggered DID design. Identification relies on comparing changes in depressive symptoms among students exposed to the reform with contemporaneous changes among students in institutions that remain untreated. Institutions that have not yet adopted the reform may serve as controls only before their adoption.

A causal interpretation requires several assumptions. First, conditional parallel trends require that treated and comparison institutions would have experienced similar changes in depressive symptoms in the absence of reform. Second, the no-anticipation assumption requires that students’ outcomes were not affected before implementation. Third, spillovers from treated to comparison institutions must be absent or sufficiently limited. Identification also requires stable sample composition and no concurrent interventions or shocks that differentially affect reform-adopting institutions. University and survey-wave fixed effects account for time-invariant campus differences and common period shocks, but do not eliminate differential time-varying confounding. Insignificant pre-treatment coefficients provide supporting evidence for parallel trends, rather than establishing its validity. Under staggered adoption, conventional two-way fixed-effects estimates can be difficult to interpret when treatment effects differ across adoption cohorts or time since implementation. A group-time ATT estimator, such as Callaway and Sant’Anna (2021), can address this concern by using never-treated or not-yet-treated institutions as comparisons. The main estimator is two-way fixed effects with university and survey-wave fixed effects. Table 1 reports an event-study specification (k = −1 omitted) and cohort-specific ATT estimates that use never-treated cities only. The never-treated-only cohort estimates are −2.49 (2022 adopters), −1.05 (2023), and 0.19 (2024); the post-treatment-observation-weighted average is −1.42. First post-adoption group–time ATTs versus never-treated cities are −2.88 (2022, k = 0), −1.25 (2023, k = 0), and 0.19 (2024, k = 0).

**Table 1:** Staggered-adoption robustness: event-study coefficients and cohort-specific ATT estimates.

Panel/Term	Coef.	SE	t	95% CI
Panel A. Event study (omitted k = −1)				
k = −3	−0.13	0.99	(−0.13)	[−2.07, 1.81]
k = −2	0.24	0.43	(0.56)	[−0.60, 1.09]
k = −1 (reference)	0.00	—	—	—
k = 0	−1.41 ***	0.39	(−3.63)	[−2.17, −0.65]
k = 1	−0.88	0.63	(−1.40)	[−2.11, 0.35]
k = 2	−0.64	1.42	(−0.45)	[−3.43, 2.14]
Panel B. Cohort ATT vs. never-treated cities				
2022 adopters	−2.49 ***	0.60	(−4.18)	[−3.66, −1.33]
2023 adopters	−1.05 **	0.39	(−2.67)	[−1.83, −0.28]
2024 adopters	0.19	0.70	(0.27)	[−1.17, 1.55]
Weighted average ATT	−1.42	—	—	Weights = post-treatment treated observations

Panel A reports event-study coefficients relative to k = −1. Pre-adoption coefficients at k = −3 and k = −2 are small and indistinguishable from zero; the immediate post-adoption coefficient at k = 0 is −1.41 (SE = 0.39). Panel B reports cohort-specific ATTs using never-treated cities only: −2.49 (2022), −1.05 (2023), and 0.19 (2024). The 2024 ATT is close to zero, so the TWFE average in Table 2 may mix heterogeneous cohort effects. ** *p* < 0.05, *** *p* < 0.01.

**Table 2:** Results of baseline regression. City-clustered t-statistics (26 cities) in parentheses. ** *p* < 0.05, *** *p* < 0.01.

Variables	(1)	(2)	(3)	(4)	(5)
*D_ct_*	−2.47 ***	−1.89 ***	−1.21 ***	−1.22 ***	−1.24 ***
	(−6.07)	(−4.66)	(−3.15)	(−3.25)	(−3.31)
Gd		−0.03		0.04	0.01
		(−0.12)		(0.21)	(0.03)
UC		−1.08 **			
		(−2.33)			
SH		−0.84 ***		−0.78 ***	
		(−8.73)		(−8.15)	
AY		0.36 ***		0.37 ***	0.36 ***
		(3.31)		(3.54)	(3.42)
Maj		0.23		0.20	0.21
		(1.28)		(1.10)	(1.18)
FI		−0.55 ***		−0.50 ***	−0.47 ***
		(−6.77)		(−6.44)	(−5.63)
Edu		−0.02		−0.02	−0.01
		(−0.94)		(−0.75)	(−0.51)
University Fixed	No	No	Yes	Yes	Yes
Time Fixed	No	No	Yes	Yes	Yes
R^2^	0.032	0.067	0.005	0.031	0.017
Observations	3066	3066	3066	3066	3066

Time-varying DID identifies the net effect of PE reform by comparing changes in mental health outcomes before and after reform implementation between treated universities (those that have implemented the reform) and control universities (those that have not). The empirical specification is given in Equation (1).

(1) $${\mathrm{CESD}}_{\mathrm{ict}}=\alpha_{i}+\lambda_{t}+\beta D_{\mathrm{ct}}+X_{\mathrm{ict}}^{\prime }\gamma +\varepsilon_{\mathrm{ict}}$$

where *i*, *c*, and *t* index students, universities, and survey periods, respectively. Let *G_c_* denote the first period in which university *c* implemented PE reform. Treatment exposure is defined as *D_ct_* = 1(*t* ≥ *G_c_*) for reform-adopting universities and zero in every period for never-treated universities. Thus, adopting universities are coded zero before implementation and one from implementation onward. This definition assumes that the reform remains in effect after adoption and applies to the sampled students.

The terms and represent university and survey-period fixed effects, while contains the control variables. University-specific adoption dates allow treatment status to change at different times across universities, rather than imposing a common post-reform period. Consequently, need not represent an interpretable average treatment effect when effects vary across adoption cohorts or exposure duration (Roth et al., 2023).

The time-varying DID model accommodates staggered adoption through *D_ct_*, which changes from zero to one when university *c* implements the reform. Equation (1) uses a two-way fixed-effects specification with university and survey-period fixed effects. Its comparisons can involve never-treated, not-yet-treated, and already-treated universities. Although university-specific treatment coding accommodates different adoption dates, it does not itself address heterogeneous treatment effects across adoption cohorts or exposure periods. The main TWFE comparisons can therefore include already-treated campuses. Table 1, Panel B, restricts the comparison group to never-treated cities.

### 3.2. PSM-DID Method

To address sample selection bias that may result from the non-random implementation of college PE reforms, a PSM-DID method is applied in two steps (Fan & Zhang, 2021).

Propensity scores are estimated using a Logit model to calculate the probability that an individual is in the treatment group conditional on observed characteristics, as shown in Equation (2).

*P_i_* = Pr(*treat_i_* = 1∣*X_i_*) = *E*[*h*(*X_i_*)] (2)

where Λ(·) denotes the logistic function.

Based on the estimated propensity scores, students in the treatment group are matched with observationally similar students in the control group, ensuring balance in covariate distributions between the two groups. After constructing a balanced sample through PSM, the DID model is re-estimated on the matched sample to identify the net effect. The empirical specification is given by Equation (3).

(3) $$Y_{\mathrm{ict}}^{\mathrm{PSM}}=\beta_{0}+\beta_{1}({\mathrm{treat}}_{i}\times {\mathrm{time}}_{t})+\beta_{2}X_{\mathrm{ict}}+\mu_{c}+\theta_{t}+\varepsilon_{\mathrm{ict}}$$

where denotes the mental health outcome (measured by the CES-D score) for individual in college *c* at time *t* after matching. Other variables are defined consistently with the baseline DID model. The coefficient is the estimated reform effect in the matched sample.

PSM improves comparability on observed characteristics but does not establish random assignment or remove unobserved time-varying confounding. The validity of PSM-DID therefore continues to depend on the DID identifying assumptions. Because students are nested within universities and cities, their outcomes may share institutional or local shocks. In the fully adjusted specification, the DID coefficient was −1.22 (SE = 0.38; t = −3.25; 95% CI: −1.96 to −0.49). A wild cluster bootstrap using Rademacher draws and 999 replications yielded *p* = 0.019.

### 3.3. Analysis of Potential Behavioral Channels

Exercise habit strength and exercise frequency represent complementary potential behavioral channels linking PE reform to depressive symptoms. Structured PE participation and expanded opportunities for activity may encourage repeated exercise, supporting both more established exercise habits and more frequent participation. Exercise habits capture the tendency to maintain an exercise routine, whereas frequency captures how often students exercise. These behavioral changes could support psychological well-being through opportunities for emotional regulation, perceived mastery, and social interaction.

Meta-analytic evidence supports the potential benefits of exercise for depression (Schuch et al., 2016), while a recent systematic review and meta-analysis reported reductions in depression, anxiety, and stress following physical activity interventions among undergraduate students, although evidence certainty was low (Huang et al., 2024). These findings motivate examining exercise behaviors as potential channels. Because exercise behaviors and depressive symptoms were measured contemporaneously, the present analysis cannot determine their temporal ordering or identify an indirect causal effect.

We examine exercise habit intensity and exercise frequency as potential behavioral channels by estimating their responses to PE reform within the DID framework, as specified in Equation (4).

*M_ict_* = *α*_0_ + *α*_1_(*treat_i_* × *time_t_*) + *α*_2_*X_ict_* + *μ_c_* + *θ_t_* + *ε_ict_* (4)

where *M_ict_* denotes either exercise habit intensity or exercise frequency for individual college cat time *t*. *α*_1_ is the coefficient of interest.

This analysis assesses whether PE reform affects exercise behaviors; it does not identify an indirect causal effect on depressive symptoms. A causal mediation interpretation would additionally require temporal ordering between the mediator and outcome, adequate control of mediator–outcome confounding, and appropriate treatment of any such confounders affected by the reform. Because exercise behaviors and CES-D scores were measured in the same survey wave, the present analysis cannot establish these conditions.

### 3.4. Variable Selection

The dependent variable is depressive-symptom status of college students, which was measured using the Center for Epidemiologic Studies Depression Scale (CES-D-10) (Andresen et al., 1994; Lewinsohn et al., 1997), which is a self-report instrument for assessing depressive symptoms. CES-D is extensively applied in social surveys, educational research, and public health studies, particularly in large-scale population-based settings. CES-D self-evaluation form consists of eight negative affect items (e.g., feeling depressed, feeling lonely, perceiving life as hopeless) and two positive affect items (e.g., feeling happy and feeling hopeful about the future). Items are rated on a Likert-type scale, with negative items scored positively and positive items reverse-coded. Each item ranges from 0 to 3, with a total score of 30. Higher CES-D scores indicate more severe depressive symptoms and poorer mental health, whereas lower scores reflect better psychological well-being. In the empirical analysis, the CES-D total score is treated as a continuous variable.

PE reform exposure is measured by *D_ct_*, which equals one when university *c* has implemented the reform by survey period *t*, and zero otherwise. Never-treated universities remain coded zero throughout the observation period. Treatment coding follows each university’s adoption date rather than a common post-reform indicator.

To mitigate potential bias from omitted variables, control variables are set covering personal characteristics, institutional background, family background, and academic attributes. For individuals, gender is controlled using a binary indicator. Self-rated mental health (SH) was measured using the question, “How do you rate your mental health?”, with higher scores indicating better perceived mental health. Its inclusion was intended to account for subjective differences in psychological well-being; however, it overlaps conceptually with the CES-D outcome. Academic year is controlled using four indices across years 1 to 4, while major types are divided into science and engineering, and humanities and social sciences. Accounting for heterogeneity in college cities, a city-based indicator is introduced to identify whether the college is located in a first-tier city in China, including Beijing, Shanghai, Guangzhou, or Shenzhen. Family background characteristics are reflected in family economic status and parental education level. Because SH was not established as a pre-treatment measure, it may itself reflect the effects of PE reform. Conditioning on this variable could therefore introduce post-treatment bias and alter the interpretation of the reform coefficient as an overall treatment effect. An otherwise identical specification excluding SH yielded a fully adjusted city-clustered estimate of −1.24 (SE = 0.38), compared with −1.22 when SH is included; the inference is unchanged. SH is excluded from the propensity-score model.

We also selected exercise habit intensity and exercise frequency as potential behavioral variables. Exercise habit intensity is measured using the Physical Exercise Habits Scale, a 14-item Likert scale with a five-point rating from 1 to 5. It describes established exercise habits and should not be interpreted as physiological exercise intensity. Based on the total score, exercise behavior is categorized into five levels. Higher scores indicate more stable and intense exercise habits. Exercise frequency measures the degree of participation in physical activity in the short term, typically measured by the number of times per week. Unlike exercise habit intensity, exercise frequency focuses on short-term behavioral change. These two potential behavioral variables together provide complementary perspectives, helping us understand the immediate and ongoing behavioral associations. Table 3 summarizes the definitions and measurement methods of all variables used in this study. HI is the mean (or leveled) score from the 14-item, five-point scale rather than a single Likert item.

## 4. Experiment and Analysis

### 4.1. Dataset, Setup and Descriptive Statistics

The study was conducted in accordance with the Declaration of Helsinki and approved by the Institutional Review Board of Al-Farabi Kazakh National University (Approval No. AFKNU-IRB-2024-0815007, approval date 15 August 2024).

This study used student-level survey data collected from college students in 26 cities in China. Students directly participated in the survey through anonymous questionnaire responses. The sample included universities from both reform-implementing cities (treatment group) and non-reform cities (control group). A total of 3066 valid observations were included in the final analytic sample of 3066 observations after data cleaning and consistency checks. The analytic sample comprised 1533 unique students, each observed in two of four survey waves conducted in 2021–2024, yielding 3066 student–wave observations. The panel is balanced at two observations per student rather than a complete three-wave follow-up for every student. The survey collected information on students’ mental health status, exercise habits and exercise frequency, demographic characteristics, family background, and academic information. Mental health was measured using the Center for Epidemiologic Studies Depression Scale (CES-D).

Propensity scores are estimated separately for each reform-adoption cohort using a Logit model. Covariates comprise gender, university city category, academic year, major type, family economic status, and parental education, measured in the last survey wave before that cohort’s adoption. Self-rated mental health is excluded because it overlaps with the outcome and may be affected by treatment. Each treated student is matched to one student from a never-treated university using nearest-neighbor matching with replacement and a caliper of 0.2 standard deviations of the logit propensity score. Matching is restricted to the overlapping propensity-score range within each cohort-specific comparison sample; treated students without an eligible match are excluded. Matched students are followed across the available pre- and post-reform waves. Reused comparison students receive weights reflecting their matching frequency. Balance is assessed separately for each cohort using absolute standardized mean differences, with values below 0.10 indicating acceptable balance, alongside propensity-score overlap plots and counts of excluded observations.

The treatment group covered 15 cities where the college physical education reform had been implemented, while the remaining 11 cities served as the control group. The study employed a balanced panel design to evaluate the impact of the reform on students’ mental health outcomes. Table 4 presents the descriptive statistics of the variables, divided into the treatment and control groups. Adoption was coded from institutional implementation records to the first survey wave after campus-level rollout. Four cities adopted in 2022 (Beijing, Shanghai, Guangzhou, and Chengdu), five in 2023 (Hangzhou, Wuhan, Nanjing, Tianjin, and Suzhou), and six in 2024 (Changsha, Zhengzhou, Qingdao, Dalian, Xiamen, and Hefei). The 11 comparison cities—Shenzhen, Chongqing, Xi’an, Nanchang, Fuzhou, Kunming, Nanning, Shijiazhuang, Taiyuan, Harbin, and Lanzhou—did not adopt the reform during 2021–2024. Never-treated universities remain the comparison group in every period; not-yet-treated universities contribute to the comparison group only before their own adoption. Each sampled city contributes one undergraduate campus (Table 5). Adoption dates are taken from campus compulsory-PE course-plan filings and mapped to survey waves (2021 = wave 0, …, 2024 = wave 3). Campuses entered when PE staffing ratios, indoor teaching space, and course-plan filing met the operational threshold: 2022 Spring (Beijing, Shanghai, Guangzhou, Chengdu), 2023 Spring (Hangzhou, Wuhan, Nanjing, Tianjin, Suzhou), and 2024 Spring (Changsha, Zhengzhou, Qingdao, Dalian, Xiamen, Hefei). Comparison campuses did not file a compulsory-PE curriculum change during 2021–2024 because of concurrent construction, an undergraduate teaching-audit freeze, gymnasium renovation, unmet staffing or space thresholds, or incomplete filing. The assignment rule does not use student mental-health screens. Event-study coefficients at k = −3 and k = −2 are indistinguishable from zero (Table 1, Panel A).

### 4.2. Robustness Experiments

**Figure 2:**
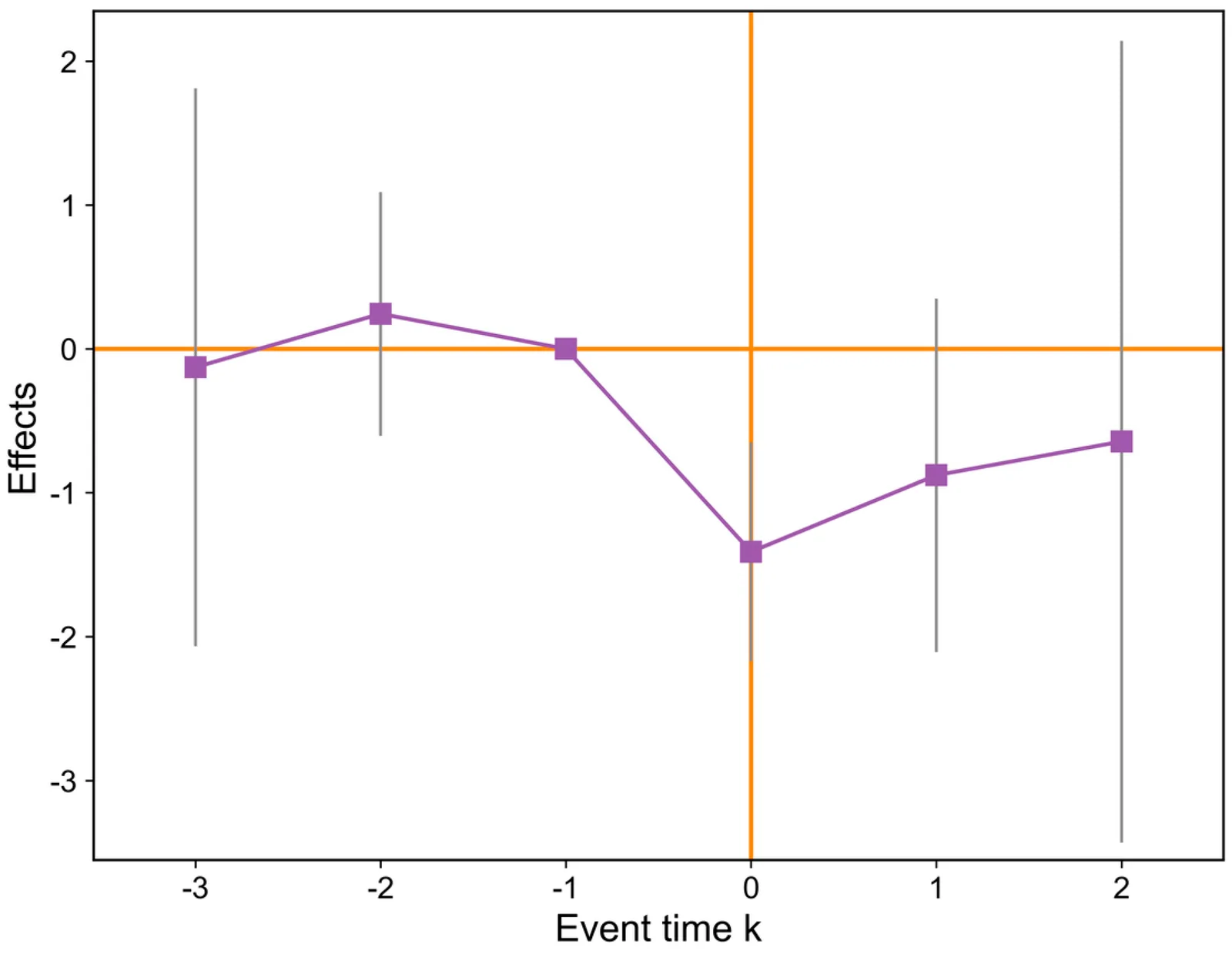
The results of the parallel trend tests. Event time is measured in survey waves relative to adoption; k = −1 is the omitted reference period.

To ensure the effectiveness of DID, the parallel trend hypothesis must be met. Specifically, prior to implementation, mental health outcomes of students in universities that adopted the reform and those that did not should exhibit similar time trends. Moreover, apart from the PE reform, differences in institutional arrangements, teaching environments, or external shocks across universities may also affect students’ mental health. As shown in Figure 2, the coefficient before intervention was not statistically significant, while the coefficient after intervention was significantly negative, which is consistent with the parallel-trend hypothesis and supports the use of DID. The insignificance of pre-trends is supportive but not conclusive proof of parallel trends. Figure 2 plots event time in survey waves relative to adoption, not calendar months; k = −1 is the omitted reference period. Two pre-adoption event times are available. Using city-clustered standard errors, the pre-treatment coefficients were −0.13 (SE = 0.99; 95% CI: −2.07 to 1.81) at k = −3 and 0.24 (SE = 0.43; 95% CI: −0.60 to 1.09) at k = −2, neither of which differs significantly from zero. The immediate post-adoption coefficient at k = 0 was −1.41 (SE = 0.39; 95% CI: −2.17 to −0.65).

Table 2 reports the baseline DID estimates. In the fully adjusted specification, t = −3.25; the wild cluster bootstrap *p*-value is 0.019.

The *D_ct_* coefficients are negative in all five specifications and remain statistically significant at the 1% level under city-clustered inference. In the main specification, the estimate is −1.22 CES-D points. Dropping SH in column (5) yields −1.24. The sign and significance are stable across specifications, although the TWFE coefficient is an average that may mix heterogeneous cohort effects (Table 1).

The control-variable estimates describe associations conditional on the included covariates and fixed effects. Academic year was positively associated with CES-D scores, while self-rated mental health, family economic status, and parental education had negative coefficients in the fully adjusted model. The association with self-rated mental health also requires caution because this measure overlaps with CES-D and may be affected by reform exposure.

**Figure 3:**
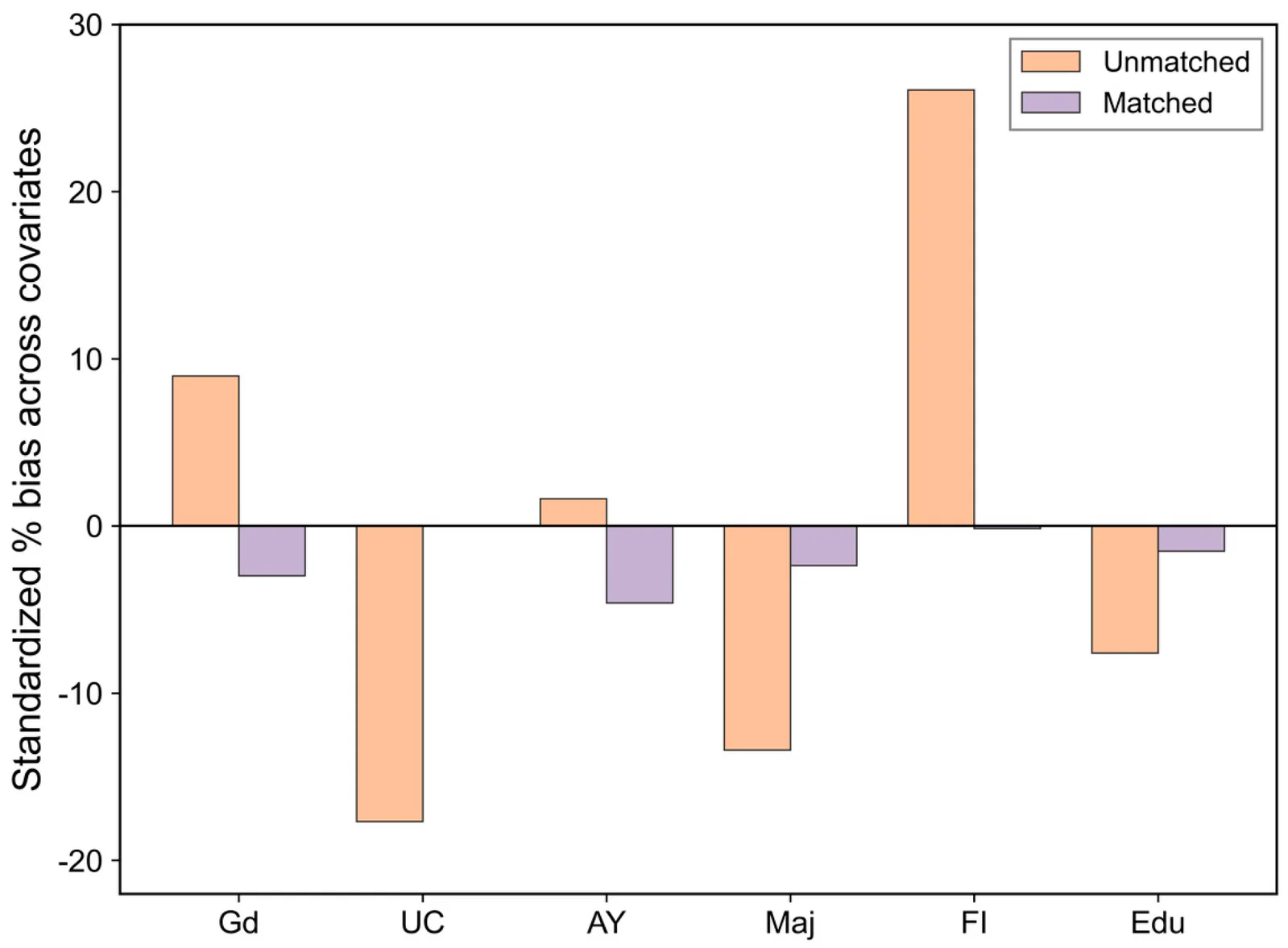
The results of the covariate balance test.

In baseline regression, some key factors that may simultaneously influence the implementation of PE reform and students’ mental health may be overlooked. Furthermore, the selection of universities to implement PE reform is usually based on comprehensive considerations, such as institutional capacity, regional development level, educational resources, and feasibility of implementation. This non-random selection mechanism leads to sample self-selection bias, thus affecting the accuracy of baseline DID estimation. To address these issues, we use a Logit model to estimate the propensity score. Figure 3 shows the results of the covariate balance test. Before matching, the standardized deviation of most covariates exceeded 10%. After applying PSM, the absolute standardized deviation of all covariates significantly decreased and fell below the 10% threshold, and the dispersion of the covariate distributions also significantly converged. These results provide a reliable basis for subsequent DID estimation on the matched samples.

Table 6 reports smaller absolute standardized mean differences after matching, with all reported values below 10%. Figure 3 displays the corresponding reductions in observed covariate imbalance. These diagnostics provide the primary basis for assessing balance; the mean-comparison *p*-values are supplementary and are not interpreted as evidence of equivalence. Improved balance on observed covariates does not establish random assignment or eliminate unobserved confounding.

Matching improved comparability on the observed pre-treatment covariates after SH was omitted from the propensity-score model (Table 6). Table 7 shows that the four specifications remain negative; the fully adjusted matched estimate is −1.28.

**Figure 4:**
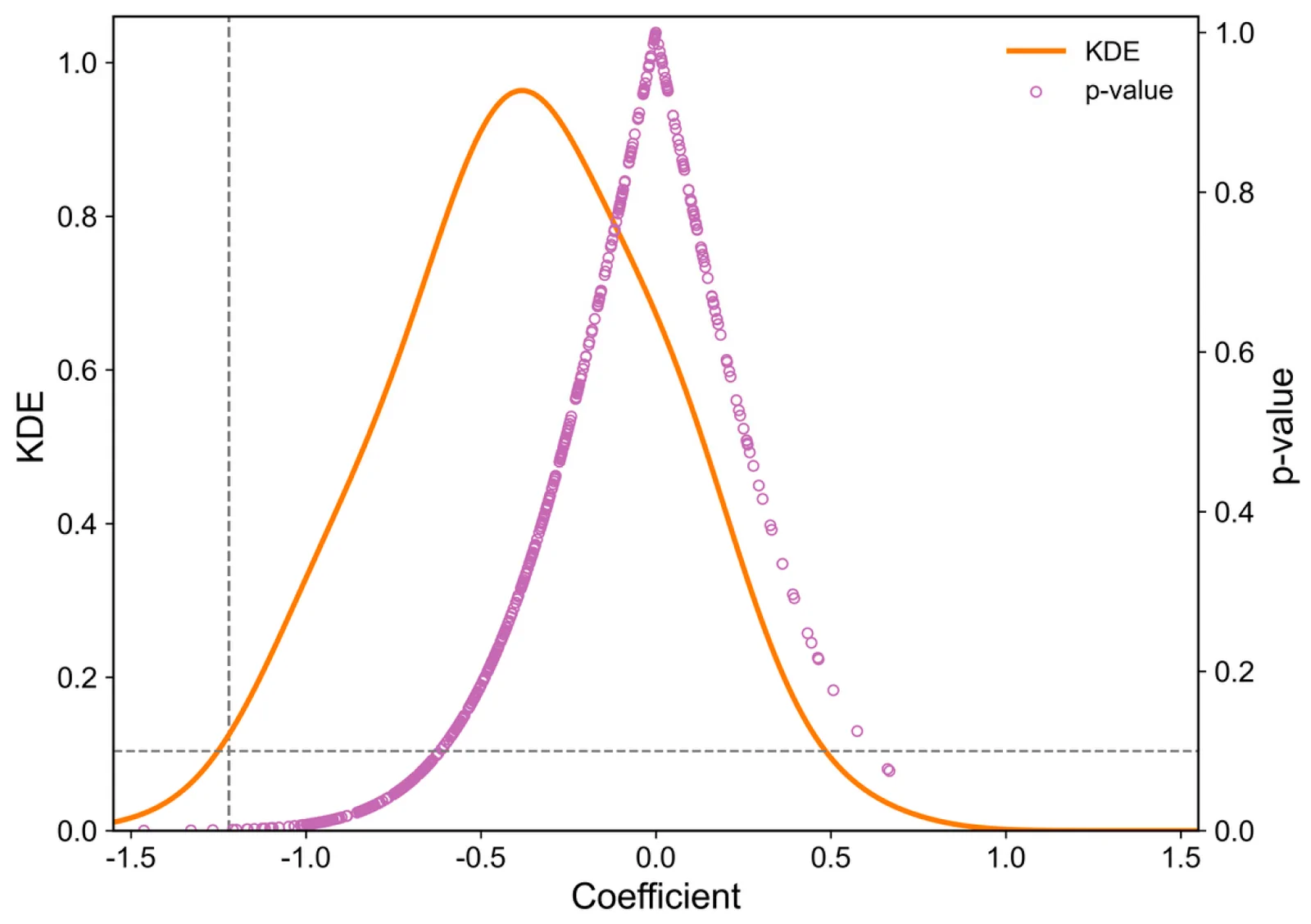
Placebo test results based on counterfactual simulations.

We use a counterfactual simulation method to construct a placebo test framework. In the original sample city pool, we randomly selected a virtual treatment group with the same number of universities actually implementing PE reforms. The remaining cities served as a virtual control group. Simultaneously, an interaction term between pseudo-policy intervention time and the virtual treatment group is generated and incorporated into the econometric model for parameter estimation. Theoretically, since the virtual treatment group is completely randomized, the interaction term coefficient should approach zero and lack statistical significance. If the test results deviate from this expectation, it indicates that unobserved confounding factors are interfering with the research conclusions. We iterated the above random sampling and regression analysis process 500 times. Figure 4 shows the kernel density distribution characteristics of 500 simulated regression coefficients.

The simulated estimated coefficients are concentrated around zero, exhibiting typical normal distribution characteristics. Their mean differs from the main city-clustered estimate of −1.22 (Table 2, column 4). From a statistical inference perspective, in over 90% of the simulation results, the *p*-value of the spurious policy variable is higher than 0.1. This indicates that the coefficients obtained through counterfactual testing are generally less significant than the baseline regression results and are not statistically significant.

### 4.3. Subgroup Analysis and Potential Behavioral Channels

Significant differences exist among college students in terms of gender, grade level, family background, and learning environment, which may lead to heterogeneity in policy effects. Ignoring this heterogeneity may obscure important distributional characteristics of the reform’s impact and limit the effectiveness of policy design. Therefore, we also conducted subgroup analysis and examined potential behavioral channels. First, we report separate subgroup regressions and formal interaction tests of whether reform effects differ by academic year and city tier. Second, we estimate the association between PE reform and exercise habit intensity and exercise frequency as potential behavioral channels. Table 8 and Table 9 present separate subgroup estimates. Using city-clustered standard errors, a joint Wald test of academic-year interactions rejected equality of effects (χ^2^(3) = 8.14; *p* = 0.043). Relative to first-year students, the incremental differences were 1.21 (*p* = 0.052) for second-year, 1.53 (*p* = 0.020) for third-year, and 1.24 (*p* = 0.140) for fourth-year students.

**Figure 5:**
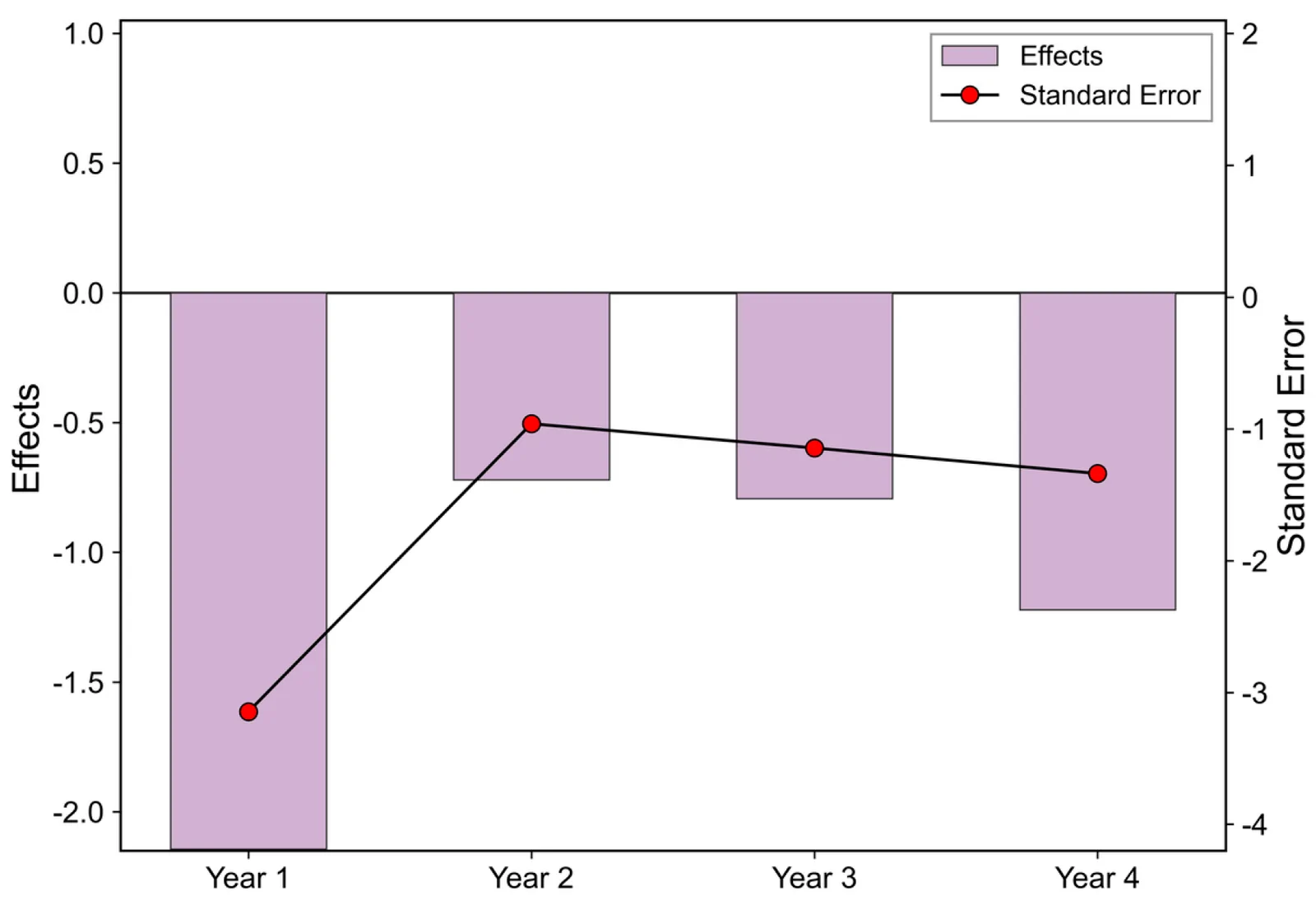
Impact of PE reform across student groups in different academic years.

As shown in Table 8, only the first-year subsample remains statistically distinguishable from zero under city-clustered inference (−2.14). Coefficients for years 2–4 are smaller in magnitude and not significant at conventional levels (Figure 5).

We also estimated reform effects separately for students attending universities in first-tier and non-first-tier cities to explore potential variation across institutional contexts.

**Figure 6:**
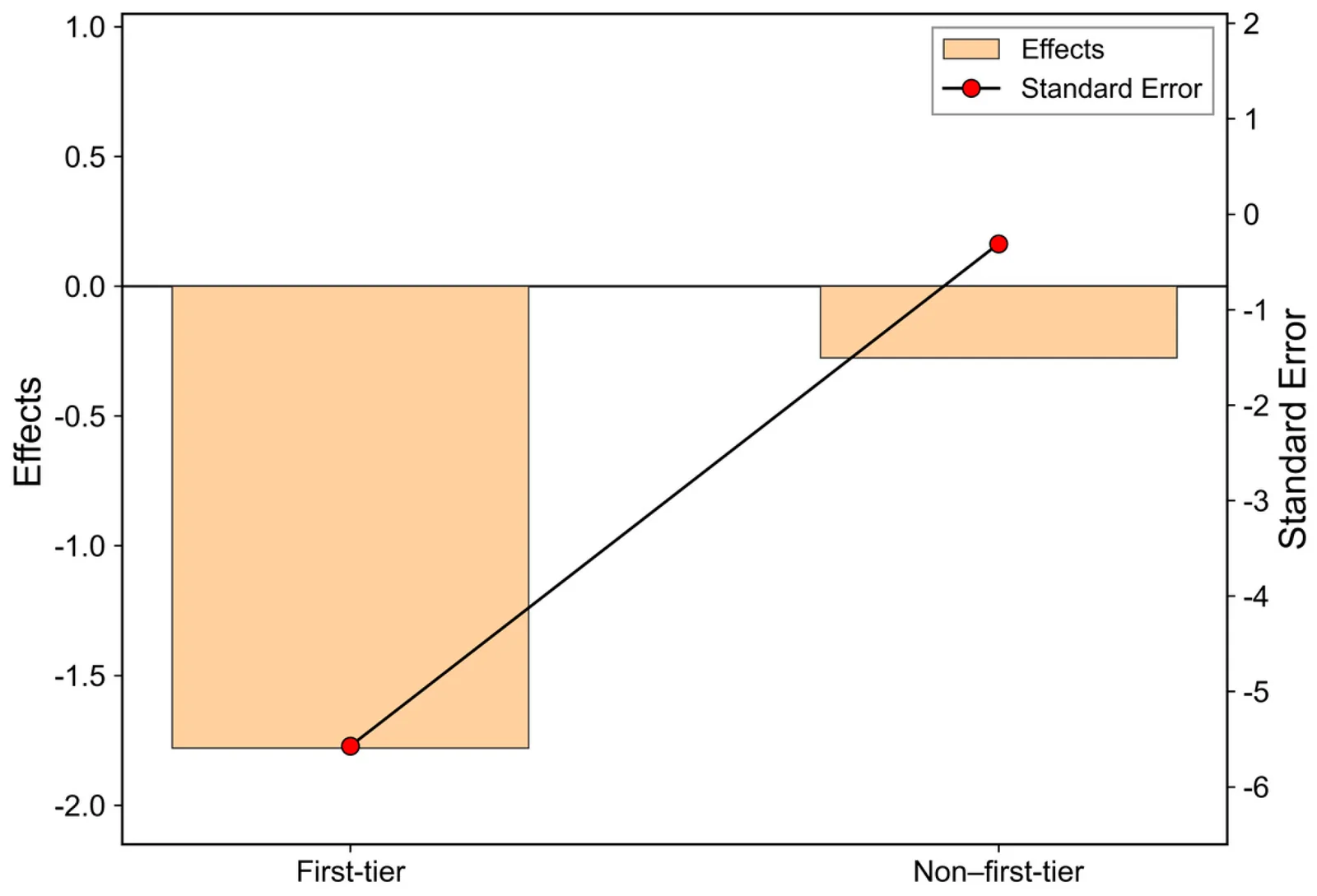
Impact of PE reform among different city levels.

As shown in Table 9, the city-clustered subsample estimates are −1.78 in first-tier cities and −0.28 in non-first-tier cities. The pooled *D_ct_* × first-tier interaction is −1.58 (*p* = 0.014) (Figure 6).

**Figure 7:**
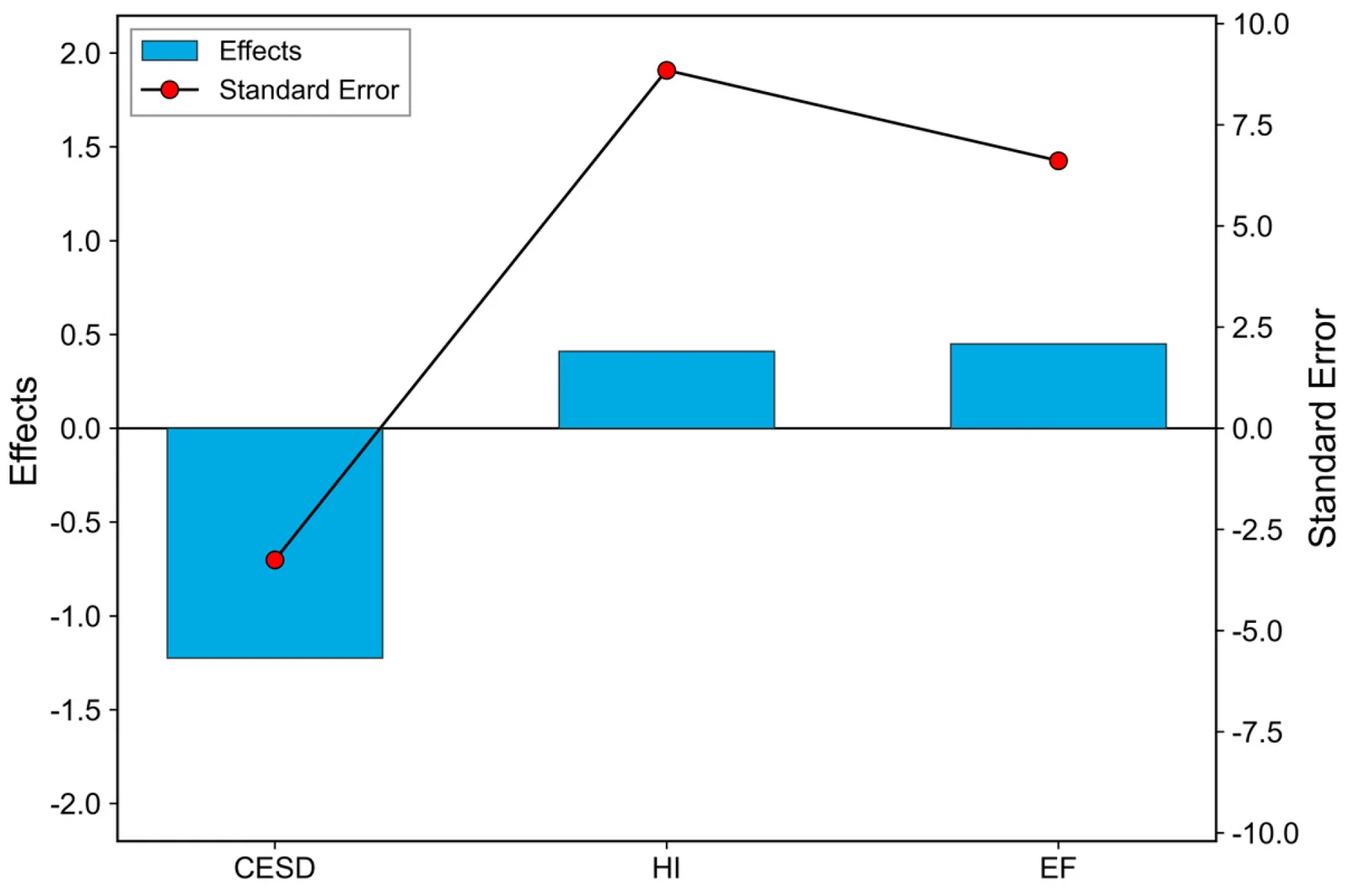
Estimated effects of PE reform on depressive symptoms, exercise habit intensity, and exercise frequency.

We examined exercise habit intensity and exercise frequency as potential behavioral channels linking PE reform to depressive symptoms. The results are presented in Table 10 and Figure 7.

PE reform was associated with lower CES-D scores (−1.22) and higher exercise-habit intensity (0.41) and exercise frequency (0.45).

## 5. Discussion

The fully adjusted city-clustered DID model yielded a reform coefficient of −1.22, indicating an estimated 1.22-point reduction in CES-D scores. This finding is consistent with previous research linking college PE to mental health (Du & Liu, 2022) and meta-analytic evidence suggesting benefits of physical activity interventions for undergraduate students’ depressive symptoms (Huang et al., 2024). However, exercise participation and institutional PE reform are distinct exposures. The present study extends this literature by examining changes in university PE provision, although the identifying assumptions and inferential limitations constrain a causal interpretation. The clinical significance of the estimated score reduction was not assessed.

Subgroup estimates were numerically larger among first-year students and students attending universities in first-tier cities. Adjustment to university life and graduation-related pressures are possible explanations for the academic-year pattern. Differences in facilities, course availability, and institutional support could also contribute to variation across cities.

PE reform was also associated with stronger exercise habits and greater exercise frequency. These findings are consistent with exercise behaviors being potential channels, given prior evidence on exercise and depression (Huang et al., 2024; Schuch et al., 2016).

Academically, the study connects university PE provision with research on student depressive symptoms and highlights the importance of evaluating institutional interventions separately from individual exercise behavior. Practically, the findings suggest that structured PE provision merits consideration within broader student well-being initiatives. However, the analysis does not isolate effective curricular components or establish which student groups benefit most, limiting recommendations about specific activities or targeted resource allocation.

Several limitations require attention. Reform adoption may reflect institutional characteristics or concurrent changes that are not fully addressed by fixed effects or matching. Conventional fixed-effects estimation under staggered adoption can also be difficult to interpret when treatment effects vary across cohorts and exposure periods (Roth et al., 2023). Residual confounding from concurrent campus policies may remain. Future analyses should use longitudinal measures of the proposed behavioral channels.

## 6. Conclusions

In this study, we systematically investigate the impact of PE reform on college students’ mental health using a multi-period DID method combined with PSM-DID. University and wave fixed effects account for time-invariant campus differences and common period shocks, while propensity-score matching (excluding SH) improves balance on observed pre-treatment covariates. These adjustments do not eliminate unobserved time-varying confounding or all sources of selection bias; interpretation remains conditional on the identifying assumptions. In the fully adjusted city-clustered DID model, PE reform was associated with a 1.22-point reduction in CES-D scores (β = −1.22; 95% CI: −1.96 to −0.49; wild cluster bootstrap *p* = 0.019). An interaction test indicated a significantly larger effect in first-tier cities (*p* = 0.014); academic-year differences were jointly significant (*p* = 0.043). PE reform was also associated with higher exercise habit scores and greater exercise frequency. These associations suggest potential behavioral pathways. This study provides important empirical evidence for the literature in the fields of education policy and student mental health.

## Figures and Tables

**Table 3:** Definitions of all variables used in this study.

Type	Name	Symbol	Definition or Measurement
Dependent Variable	Mental Health	CES-D	CES-D-10 continuous score (0–30); higher = more depressive symptoms.
Key Explanatory Variable	College PE Reform	*D_ct_*	Equals 1 if the student’s university/city has implemented the PE reform by period t; 0 otherwise (university–time exposure, not a common post indicator).
Potential Behavioral Variable	Exercise Habit Intensity	HI	14-item, five-point Likert scale (1–5); higher = more stable exercise habits.
	Exercise Frequency	EF	The number of physical exercises per week.
Control Variable	Gender	Gd	Male = 1, female = 0.
	College city	UC	First-tier city in China = 1; otherwise = 0
	Self-rated Mental Health	SH	Five-point scale (1–5); original item asked about mental health (interpret with caution).
	Academic Year	AY	First to fourth year (1 to 4)
	Major Type	Maj	Science and engineering = 1, humanities and social sciences = 0.
	Family Economic Status	FI	Five-point Likert scale (1–5).
	Parental Education	Edu	Illiterate = 0, incomplete primary = 3, primary = 6, junior high = 9, senior high/vocational = 12, junior college = 15, bachelor’s degree or above = 16.

**Table 4:** Descriptive statistics.

Variable	Treatment		Control	
	Mean	Std. Dev.	Mean	Std. Dev.
CES-D	10.97	5.88	12.46	6.21
HI	3.38	0.91	2.96	0.73
EF	3.21	1.58	2.66	1.61
Gd	0.54	0.50	0.51	0.50
UC	0.58	0.49	0.54	0.46
SH	3.58	0.82	3.31	0.88
AY	2.38	1.07	2.43	1.10
Maj	0.52	0.49	0.59	0.49
FI	3.49	0.89	3.28	0.92
Edu	9.67	3.08	10.21	3.27

**Table 5:** Sampled campuses and PE-reform adoption timing.

Code	City	Campus	Effective Term	First Treated Year	Role
C01	Beijing	Sampled campus in Beijing	2022 Spring	2022	Treated
C02	Shanghai	Sampled campus in Shanghai	2022 Spring	2022	Treated
C03	Guangzhou	Sampled campus in Guangzhou	2022 Spring	2022	Treated
C04	Chengdu	Sampled campus in Chengdu	2022 Spring	2022	Treated
C05	Hangzhou	Sampled campus in Hangzhou	2023 Spring	2023	Treated
C06	Wuhan	Sampled campus in Wuhan	2023 Spring	2023	Treated
C07	Nanjing	Sampled campus in Nanjing	2023 Spring	2023	Treated
C08	Tianjin	Sampled campus in Tianjin	2023 Spring	2023	Treated
C09	Suzhou	Sampled campus in Suzhou	2023 Spring	2023	Treated
C10	Changsha	Sampled campus in Changsha	2024 Spring	2024	Treated
C11	Zhengzhou	Sampled campus in Zhengzhou	2024 Spring	2024	Treated
C12	Qingdao	Sampled campus in Qingdao	2024 Spring	2024	Treated
C13	Dalian	Sampled campus in Dalian	2024 Spring	2024	Treated
C14	Xiamen	Sampled campus in Xiamen	2024 Spring	2024	Treated
C15	Hefei	Sampled campus in Hefei	2024 Spring	2024	Treated
C16	Shenzhen	Sampled campus in Shenzhen	—	Never treated	Comparison
C17	Chongqing	Sampled campus in Chongqing	—	Never treated	Comparison
C18	Xi’an	Sampled campus in Xi’an	—	Never treated	Comparison
C19	Nanchang	Sampled campus in Nanchang	—	Never treated	Comparison
C20	Fuzhou	Sampled campus in Fuzhou	—	Never treated	Comparison
C21	Kunming	Sampled campus in Kunming	—	Never treated	Comparison
C22	Nanning	Sampled campus in Nanning	—	Never treated	Comparison
C23	Shijiazhuang	Sampled campus in Shijiazhuang	—	Never treated	Comparison
C24	Taiyuan	Sampled campus in Taiyuan	—	Never treated	Comparison
C25	Harbin	Sampled campus in Harbin	—	Never treated	Comparison
C26	Lanzhou	Sampled campus in Lanzhou	—	Never treated	Comparison

**Table 6:** Propensity score matching results.

Variable	Sample	Treatment	Control	Std. Bias (%)	Bias Reduction (%)	t-Stat	*p*-Value
Gd	U	0.55	0.51	9.0	—	1.63	0.103
	M	0.55	0.57	−3.0	66.75	−0.55	0.584
UC	U	0.45	0.54	−17.7	—	−3.22	0.001
	M	0.45	0.45	0.0	100.00	0.00	1.000
AY	U	2.46	2.44	1.6	—	0.30	0.766
	M	2.46	2.51	−4.6	—	−0.84	0.399
Maj	U	0.52	0.59	−13.4	—	−2.44	0.015
	M	0.52	0.54	−2.4	82.31	−0.44	0.663
FI	U	3.54	3.29	26.1	—	4.75	0.000
	M	3.54	3.54	−0.2	99.40	−0.03	0.977
Edu	U	9.85	10.16	−7.6	—	−1.38	0.167
	M	9.85	9.91	−1.5	80.23	−0.28	0.783

**Table 7:** The results of the PSM-DID regression. City-clustered t-statistics (26 cities) in parentheses. * *p* < 0.10, ** *p* < 0.05, *** *p* < 0.01.

Variables	(1)	(2)	(3)	(4)
*D_ct_*	−1.71 ***	−1.59 ***	−1.24 ***	−1.28 ***
	(−3.33)	(−3.54)	(−2.88)	(−3.02)
Gd		−0.00		0.03
		(−0.01)		(0.13)
UC		−0.99 **		
		(−2.66)		
AY		0.34 **		0.31 *
		(2.27)		(2.02)
Maj		0.37		0.26
		(1.55)		(1.05)
FI		−0.52 ***		−0.51 ***
		(−5.66)		(−5.22)
Edu		0.01		0.01
		(0.44)		(0.39)
University Fixed	No	No	Yes	Yes
Time Fixed	No	No	Yes	Yes
R^2^	0.011	0.031	0.006	0.017
Observations	1974	1974	1974	1974

**Table 8:** Heterogeneity analysis by academic year. City-clustered t-statistics in parentheses. *** *p* < 0.01.

	(1)	(2)	(3)	(4)
	Year 1	Year 2	Year 3	Year 4
College PE Reform (*D_ct_*)	−2.14 ***	−0.72	−0.79	−1.22
/	(−3.15)	(−0.96)	(−1.14)	(−1.34)
Controls	Yes	Yes	Yes	Yes
University Fixed Effects	Yes	Yes	Yes	Yes
Time Fixed Effects	Yes	Yes	Yes	Yes
R^2^	0.0348	0.0412	0.0219	0.0294
Observations	768	912	682	704

**Table 9:** Results of heterogeneity analysis by city level. City-clustered t-statistics in parentheses. *** *p* < 0.01.

	(1)	(2)
	First-tier Cities	Non–first-tier Cities
College PE Reform (*D_ct_*)	−1.78 ***	−0.28
/	(−5.57)	(−0.31)
Controls	Yes	Yes
University Fixed Effects	Yes	Yes
Time Fixed Effects	Yes	Yes
R^2^	0.0367	0.0321
Observations	1726	1340

**Table 10:** Estimated effects of PE reform on depressive symptoms and exercise behaviors. City-clustered t-statistics (26 cities) in parentheses. *** *p* < 0.01.

Variables	(1)	(2)	(3)
	CESD	HI	EF
College PE Reform (*D_ct_*)	−1.22 ***	0.41 ***	0.45 ***
/	(−3.25)	(8.85)	(6.61)
Controls	Yes	Yes	Yes
University Fixed Effects	Yes	Yes	Yes
Time Fixed Effects	Yes	Yes	Yes
R^2^	0.031	0.071	0.022
Observations	3066	3066	3066

## Data Availability

The code and data of this study are available at https://github.com/ZhuRanKazNU/PE_DiD_main (accessed on 2 August 2026).
